# Wanderer, an interactive viewer to explore DNA methylation and gene expression data in human cancer

**DOI:** 10.1186/s13072-015-0014-8

**Published:** 2015-06-23

**Authors:** Anna Díez-Villanueva, Izaskun Mallona, Miguel A. Peinado

**Affiliations:** Institute of Predictive and Personalized Medicine of Cancer (IMPPC), Ctra. de Can Ruti, camí de les Escoles, s/n, 08916 Badalona, Spain; Health Research Institute Germans Trias i Pujol (IGTP), Can Ruti Campus, Ctra. de Can Ruti, camí de les Escoles, s/n, 08916 Badalona, Spain

## Abstract

**Background:**

The Cancer Genome Atlas (TCGA) offers a multilayered view of genomics and epigenomics data of many human cancer types. However, the retrieval of expression and methylation data from TCGA is a cumbersome and time-consuming task.

**Results:**

Wanderer is an intuitive Web tool allowing real time access and visualization of gene expression and DNA methylation profiles from TCGA. Given a gene query and selection of a TCGA dataset (e.g., colon adenocarcinomas), the Web resource provides the expression profile, at the single exon level, and the DNA methylation levels of HumanMethylation450 BeadChip loci inside or in the vicinity of the queried gene. Graphic and table outputs include individual and summary data as well as statistical tests, allowing the comparison of tumor and normal profiles and the exploration along the genomic locus and across tumor collections.

**Conclusions:**

Wanderer offers a simple interface to straightforward access to TCGA data, amenable to experimentalists and clinicians without bioinformatics expertise. Wanderer may be accessed at http://maplab.cat/wanderer.

## Background

Cancer includes a group of largely heterogeneous and complex diseases. The development of massive genomic analysis techniques and the coordinated effort of different international consortiums are providing the research community with comprehensive data of tissue samples from thousands of cancer patients. Free access to these data represents an invaluable opportunity for the research community to get insights into the molecular profiles of cancers allowing, for instance, identification of new candidate markers, in silico hypothesis testing, or validation of in-house results through an independent series.

Cancer research is benefited by coordinated efforts on data acquisition and analysis. The Cancer Genome Atlas (TCGA) [1] is probably the most ambitious of these initiatives offering a multilayered view of genomics and epigenomics data together with clinicopathological information of more than 30 human cancer types. Major contributions of TCGA consortium are illustrated by the opening manuscripts (mainly devoted to report the mutational landscapes) of each one of the considered human cancers [2]; but a large number of studies are taking advantage of the available datasets [3].

TCGA Data Portal [4] is the default gateway to retrieve data offering a text file for each processed sample belonging to a dataset generated using a given platform. Compressed packages for multiple samples are allowed. For instance, it allows downloading 992 files with the clinical data related to samples of the Colon Adenocarcinoma (COAD) dataset, but only provides RNA-Seq results for 303 of them. It is worth noting that as the number of files and their sizes increase, further processing of this information exceeds the spreadsheet-based analysis capabilities and requires bioinformatics skills.

Cancer research teams working on specific aspects of the disease (e.g., exploration of candidate biomarkers, deregulation of signaling pathways, or mechanisms of gene regulation, just to name a few) might be more interested in rapidly querying specific subsets of the data (e.g., expression levels of a named gene or the methylation status of its promoter region) rather than obtaining bulky datasets as offered by the data portal. To facilitate the access and exploitation of TCGA data, several tools have been developed to compare and to explore the different levels of data, facilitating the discovery of associations and correlations, as well as allowing ascertaining the significance of certain alterations in cancer [5–7].

The high level of complexity of transcriptomic and epigenetic profiles in normal and cancer samples poses challenging scenarios to analyze, represent, and interpret both gene-specific and bulk data. Important differences among samples may be easily missed if one-size-fits-all standardized criteria are used. For instance, gene expression differences may appear as alternative transcripts and be missed in summarizing values. Moreover, relevant DNA methylation changes may occur outside predefined promoter domains or just affect a subregion.

Here we present Wanderer, a very simple and intuitive Web tool allowing real-time access and visualization of gene expression and DNA methylation profiles from TCGA data using gene targeted queries. Wanderer is addressed to a broad variety of experimentalists and clinicians without deep bioinformatics skills.

## Results

### DNA methylation profiling

**Fig. 1:**
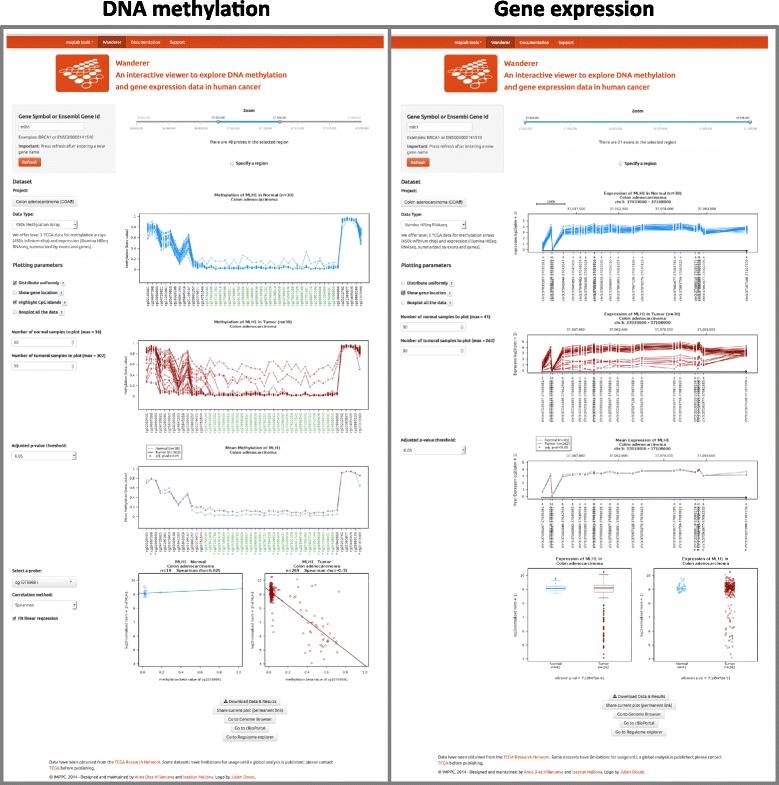
Snapshot of Wanderer Web page. Illustrative example of the DNA methylation (*left* window) and gene expression profiles (*right* window) associated with MLH1 gene in colon cancer dataset. The left panel in each window includes data selection (gene name/ID and dataset) and view customization parameters (graphics options, statistical threshold, etc.). The *right* panel of each window displays individual and summarized profiles for normal and tumor samples. The DNA methylation window allows interrogating the correlation between DNA methylation of individual probes with gene expression. At the *bottom* of the window, direct links provide data download and customized access to other Web portals containing TCGA data

For any given gene selected by the investigator, the tool provides the detailed individual beta values of all the HumanMethylation450 probes inside or in the vicinity of the gene. Graphs for normal and tumor samples from any of the available TCGA datasets are readily produced (Fig. 1). A summarizing plot is also generated, and statistical comparisons are performed to identify differential DNA methylation between normal and tumor samples at the single probe level. Wanderer performs Wilcoxon rank sum test on normal versus tumor provided there are at least two observations in each group. Although this is readily reported, we note that addressing the significance on few observations and without inspecting the data distribution might be meaningless. Even though the number of samples to draw as line plots can be modified manually (see below), the statistical analysis is performed with the complete dataset.

Wanderer complements the DNA methylation profiling with a tool to overview the influence of DNA methylation on gene expression. The user is allowed to navigate through any of the HumanMethylation450 probes located in the displayed genomic region to explore its correlation with the gene expression values.

The generated plots and comprehensive tables containing all the relevant information (individual data, statistical analyses, and additional clinical data) may be downloaded for further data analysis or representation with any software the investigator is used to (e.g., Excel or any other spreadsheet or graphing tool). In addition, the tool allows local navigation and zoom in/out within the region of interest as well as simple graph customization as described below.

### Gene expression profiling

For any selected gene, Wanderer displays the expression of each exon measured as reads per kilobase per million mapped reads (RPKM) (Fig. 1). A summarizing plot and statistical analysis is also performed to denote exons with significant differences between normal and tumor tissues. Wanderer also represents the summarized gene expression values in RSEM format (RNA-Seq by Expectation-Maximization) [8] as a boxplot and a scatterplot for normal and tumor samples. A Wilcoxon rank sum test checks whether normal and tumors are statistically equivalent.

Similarly to the DNA methylation profiling, gene expression graphs can be modified using predefined options (see below). Finally, the plots and gene expression data may be downloaded as files in compatible formats for further analysis and representation.

### Plotting versatility

Graphical outputs are highly dynamic, being rendered on the fly. Once the gene of interest is selected, some parameters can be easily tuned:

- The user can zoom in and out either by using a simple slider or entering a custom coordinate range by hand.
- A glyph depicting the actual gene location and transcription direction may be optionally shown.
- Methylation probes belonging to a CpG island can be highlighted in green.
- Distance between probes or exons can be selected to be proportional to the actual genomic location or to be uniform. In the case of methylation, this feature facilitates visualization of regions with high density of probes, such as CpG islands.
- Individual DNA methylation and gene expression profiles are drawn as line plots by default. In this representation, the scrutinized exons or CpGs belonging to the same sample are connected by lines. The user can modify the number of samples to be represented this way. A random sampling (with a random seed so the result is repeatable) is therefore performed. However, all the data can be taken into account at once ticking the “Boxplot all the data” checkbox.
- The user can select the *p* value threshold by exon or by CpG normal vs tumor comparison.

### Further capabilities

Data, analysis, and graphics may be downloaded as different files packaged in a single ZIP-compressed archive. This archive is named with a unique identifier containing the gene name, dataset, and the user’s timestamp. Graphics are provided in raster (PNG) and vector (PDF) formats. Probe annotations, statistical analysis, and clinical and raw data for both normal and tumor samples are available as text files (comma-separated values (CSV)) that can be processed using spreadsheet software. The ZIP file also includes the user manual with a detailed summary of the outputs and the software and data version.

Wanderer also offers batch analysis capabilities as well data sharing and linking from other Web resources through a Wanderer application programming interface (API) [9]. Wanderer API has the same capabilities as Wanderer but receives the parameters parsing arguments from the URL.

The tool provides deep links to explore additional information regarding the preselected gene and TCGA dataset: the cBioPortal [10] provides access to mutations, protein and survival data, among others; the All Pairs Regulome Explorer [11] presents an interactive visualization of associations between the region of interest and different types of data including mutations, copy number alterations, or clinical information; and finally, a link to the UCSC Genome Browser [12] allows a genomic view of the scrutinized region.

### Usage examples

Below we report three queries illustrating issues that can be easily resolved by Wanderer, but requiring a large number of steps and/or computational skills to be achieved by other means.

**Fig. 2:**
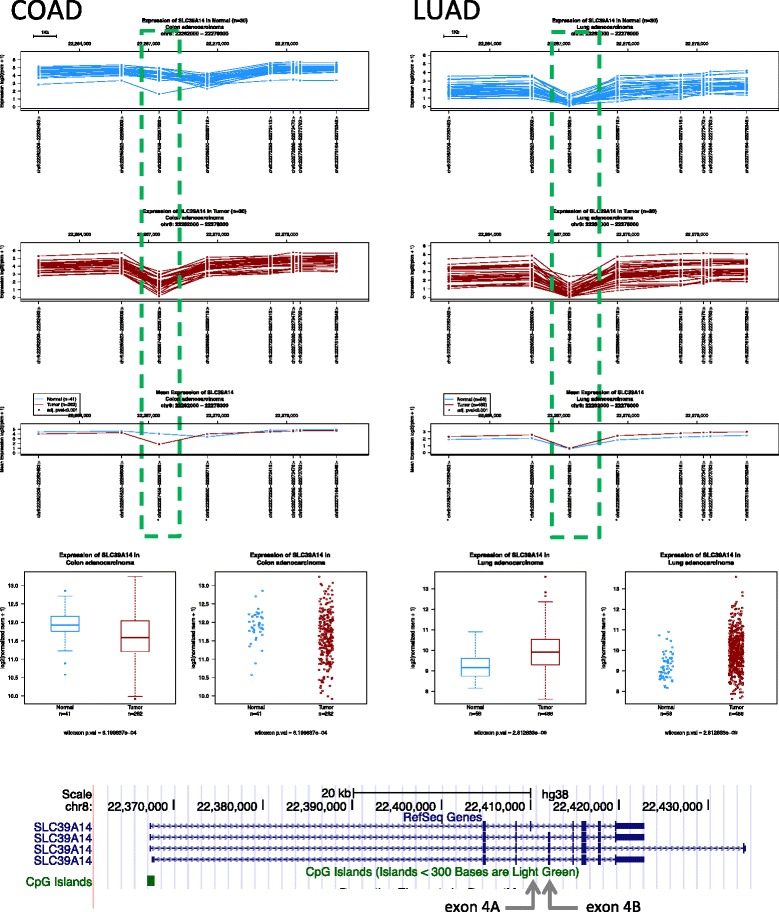
Gene expression profile of SLC39A14 in colon adenocarcinoma (COAD) and lung adenocarcinoma (LUAD). The SLC39A14 gene shows a different expression profile between normal and tumor colon, with most marked differences in the region denoted by a *box*. In contrast, lung normal and malignant tissues display similar profiles. The *bottom* panel shows SLC39A14 isoforms of in the UCSC genome browser window and the *arrows* point the location of alternatively spliced exons 4A and 4B
- DNA methylation profiles. Which region of the CpG island should I analyze to find the largest abnormal DNA methylation in tumor samples? Is it the same for different tumor types? As shown in Fig. 3a, the PTGIS CpG island is represented by five HumanMethylation450 BeadChip probes. A high proportion of colon tumors show hypermethylation of the CpG island, but the one that is farther from the TSS (denoted with an arrowhead) is already partially methylated in normal tissue and therefore is the less discriminative one. On the contrary, this position remains fully unmethylated in most normal prostate tissues, but is highly hypermethylated in prostate cancer. PTGIS is downregulated in both colon and prostate cancer (Fig. 3b).

- Gene expression profiles. Are different samples expressing the same gene isoforms? Which exon(s) should I analyze to find the largest difference between normal and tumor? As shown in Fig. 2, the abundance of the different exons of the SLC39A14 gene (also known as zinc transporter ZIP14) shows an uneven balance in normal and tumor tissues in colon cancer patients. These differences are explained by the predominance of the transcript containing exon 4A in normal colon, while cancer cells display an alternative splicing with increased representation of exon 4B [13]. Interestingly, the colon cancer profile is similar to the one present in lung (Fig. 2, right panel) and other tissues (data not shown), with no differences between normal and tumor tissues.

**Fig. 3:**
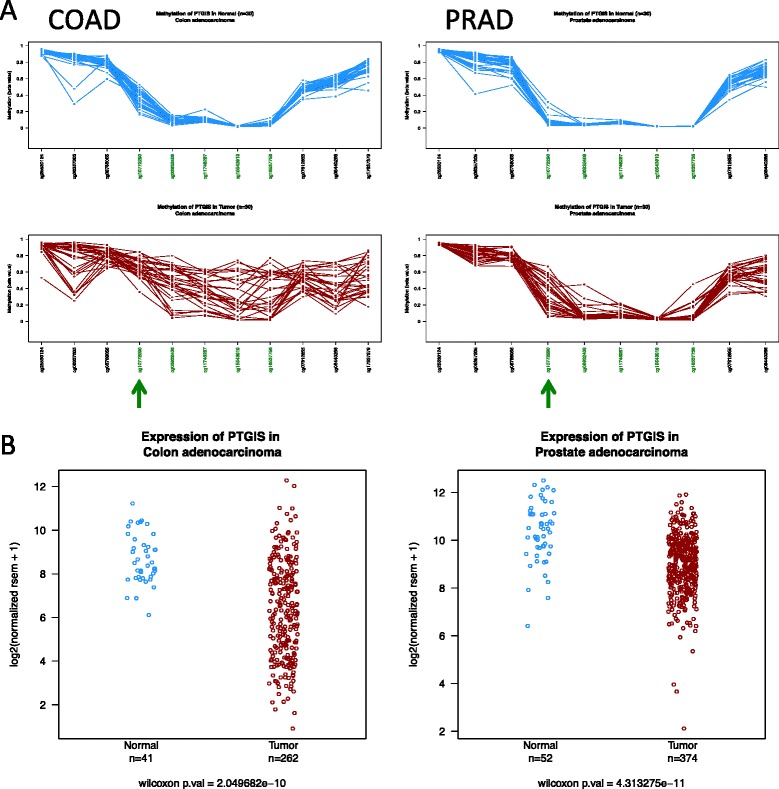
DNA methylation and gene expression of PTGIS. **a** DNA methylation profile of PTGIS CpG island in colon adenocarcinomas (COAD) and prostate adenocarcinomas (PRAD). Note that cg1077290 probe (denoted by an *arrowhead*) is already very methylated in colon normal tissue, offering poor discriminative resolution to detect tumor hypermethylation compared with the rest of CpG island probes (labeled in *green*). In contrast, this probe (cg1077290) shows the highest level of hypermethylation in prostate cancers (PRAD) and remains unmethylated in normal tissue, resulting as the most discriminant variable in this type of cancer. **b** Overall gene expression of PTGIS gene in colon and prostate samples
- DNA methylation profiles. What is the extent of DNA methylation changes in regard to a specific gene? EN1 CpG island is frequently hypermethylated in both colorectal and breast cancer (Fig. 4a). Nevertheless, when zooming out to the flanking regions (Fig. 4b), the profiles clearly show that hypermethylation affects many neighboring CpG islands in colon cancer but not in breast cancer, where hypermethylation is restricted to the EN1 CpG island (denoted by an arrowhead).

**Fig. 4:**
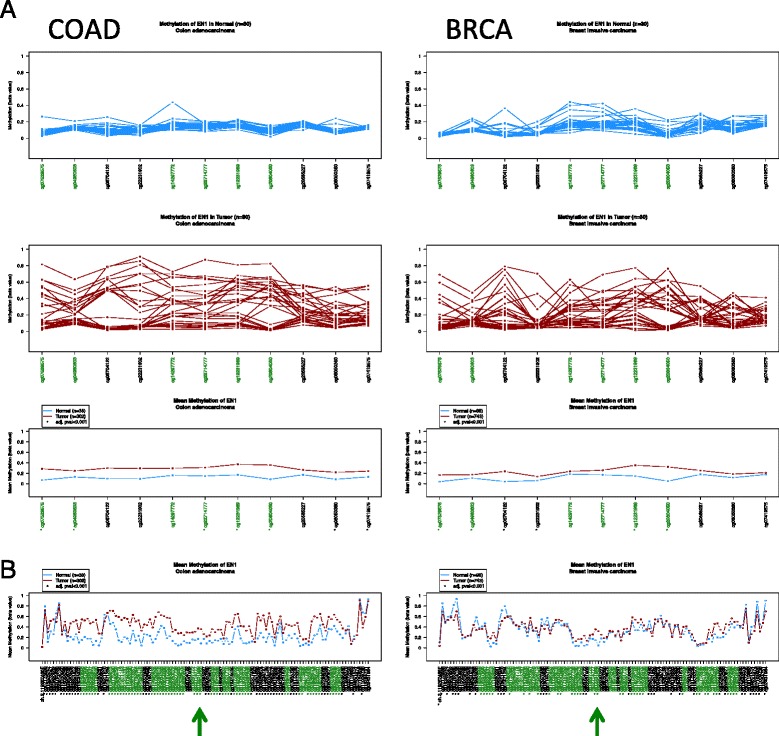
DNA methylation profile of the EN1 gene CpG island and flanking regions in colon (COAD) and breast carcinomas (BRCA). **a** Individual and mean methylation beta values of EN1 CpG island and the adjacent CpGs (chr2: 119,604,000–119,606,000). The region remains unmethylated in normal tissues and becomes hypermethylated in both colon and breast cancers. **b** Zooming out the region (chr2: 119,540,000–119,706,000) reveals the presence of a large number of CpG islands (*green* probes) and global hypermethylation of most of them in colon adenocarcinomas. Alternatively, breast cancers display interspersed regions of hypomethylation and hypermethylation. The graphs display the average methylation values of each dataset. Probes marked with an *asterisk* denote statistically significant differences between normal and tumor samples. *Arrowheads* indicate the position of the EN1 CpG island probes

## Discussion

Integration and mining of TCGA data is a hot topic in clinical bioinformatics. Given the massive nature of data, some of the tools are primarily focused on data retrieval, such as TCGA-Assembler [14], and further annotation and indexing, such as Firehose [15]. A second group of analytical platforms are devoted to provide a comprehensive view of the multiple layers of TCGA by linking molecular data to the clinical features. An outstanding example is cBioPortal [10], which delivers multiple outputs including survival analysis. Regulome Explorer [11] shows associations between clinical and molecular features and provides a circular-based visualization of the data, among others. The Cancer Genome Workbench (CGWB) [16] provides many interfaces to integrate and visualize multiple layers of data. Finally, other tools with a broader scope, as the UCSC Genome Browser, have incorporated TCGA data allowing genomic coordinate-based display [17]. A comprehensive list of cancer data visualization tools may be found at TCGA website [18] and recent reviews [5, 6].

These ambitious initiatives are invaluable contributions which facilitate the access and analysis of the vast amounts of data; but they may be deceiving to wet lab researchers and clinicians with limited computational skills and who are just willing to query very specific information. To face these needs, some tools have been developed providing a very simple interface specialized in a few layers of information. Remarkable examples are MethHC [19] and MENT [20], mainly focused on the comparison of gene expression and DNA methylation.

Wanderer offers a gene-centered access to gene expression and DNA methylation TCGA data. The most outstanding and differential feature of this tool is data display and analysis in a regional framework. The intuitive, simple, and interactive interface facilitates the visualization, interpretation, and obtention of otherwise complex information not easily reachable in other packages. Direct view of the expression and DNA methylation profiles in a regional context may have important applications as illustrated by the usage examples reported above. A quick view of the profile in a specific dataset should be extremely useful to design the most appropriate strategy (i.e., choosing the region or the probe to be analyzed) to determine either the expression of the DNA methylation of a given gene in an experimental setting. These scenarios are common in molecular studies, and the utility of the information is not limited to cancer research. Basic and clinical researchers working in any field of human biology may be also interested in exploring the features of their favorite gene, especially in normal tissues.

Another important feature provided by Wanderer is the simple interface to download small subsets of data in a format easily amenable for customized statistical and graphical analyses. Any user with average proficiency in office suites (Microsoft Office, OpenOffice, IWorks, etc.) or statistics packages (SPSS, SAS, R, etc.) can obtain the desired dataset in a compatible format by just by pressing a button.

## Conclusions

Wanderer is an intuitive interface to explore and interpret gene-associated profiles of expression and DNA methylation for all the cancer types available at TCGA. Normal-tumor paired comparisons are readily provided in the form of graphs and comprehensive tables, facilitating the selection of candidate loci to be considered in experimental and statistical settings. The outputs may be downloaded and shared via API. Wanderer is freely accessible at http://maplab.cat/wanderer.

## Methods

The user-friendly Web application has been developed using R/shiny and a PostgreSQL backend using an eXtreme programming software development methodology. Wanderer is compatible with most common Web browsers and operative systems, requires no installation, and can be run on commodity computers, such as low-memory laptops.

Data consists of HumanMethylation450 BeadChip and Illumina HiSeq exon and gene quantifications as computed by the RNA-Seq Version 2 pipeline. Data was downloaded with TCGA-Assembler [14]. Source code is available under the GPL v2 terms at [21].
